# Shared patterns in body size declines among crinoids during the Palaeozoic extinction events

**DOI:** 10.1038/s41598-021-99789-6

**Published:** 2021-10-13

**Authors:** Mariusz A. Salamon, Tomasz Brachaniec, Dorota Kołbuk, Anwesha Saha, Przemysław Gorzelak

**Affiliations:** 1 Faculty of Natural Sciences, University of Silesia in Katowice, Będzińska 60, 41-200 Sosnowiec, Poland; 2 Institute of Paleobiology, Polish Academy of Sciences, Twarda 51/55, 00-818 Warszawa, Poland

**Keywords:** Palaeontology, Evolutionary ecology, Palaeoecology

## Abstract

Crinoids were among the most abundant marine benthic animals throughout the Palaeozoic, but their body size evolution has received little attention. Here, we compiled a comprehensive database on crinoid calyx biovolumes throughout the Palaeozoic. A model comparison approach revealed contrasting and complex patterns in body size dynamics between the two major crinoid clades (Camerata and Pentacrinoidea). Interestingly, two major drops in mean body size at around two mass extinction events (during the late Ordovician and the late Devonian respectively) are observed, which is reminiscent of current patterns of shrinking body size of a wide range of organisms as a result of climate change. The context of some trends (marked declines during extinctions) suggests the cardinal role of abiotic factors (dramatic climate change associated with extinctions) on crinoid body size evolution; however, other patterns (two intervals with either relative stability or steady size increase in periods between mass extinctions) are more consistent with biotic drivers.

## Introduction

The size of an organism is undoubtedly one of its most prominent features, affecting nearly all aspects of life history, physiology, behaviour, ecology, and evolution^1^. Not surprisingly, body size is commonly under strong selection pressure; therefore, its evolution is a central focus of evolutionary studies^2,3^. Despite long-standing interests in such research, trends in body size of some major marine animal clades and their underlying evolutionary drivers still remain poorly understood.

Crinoids (Crinoidea), commonly called sea lilies or feather stars, were among the most dominant components of Palaeozoic benthic palaeocommunities. For instance, the Mississippian period is commonly referred to as the “Age of Crinoids”^4^ because of their outstanding high diversity and abundance. Palaeozoic crinoids, due to their high fossilisation potential and a densely sampled fossil record^5–7^, present an ideal model for studying long-term body size evolution. Surprisingly, however, though considerable effort has been devoted to examining their diversity and disparity patterns^5–11^, studies exploring large‐scale body-size trends in fossil crinoids are almost lacking. Although a few relevant datasets are available, they either span only few geological stages^12^, focus on a local scale and/or within lineage size variation of a specific clade^13^, or provide global coverage but are hampered by inclusion of fragmentary and out-of-date source of data and lack critical and comprehensive analysis^1^. The present paper aims to fill this gap by investigating macroevolutionary body-size trends of crinoids across the Palaeozoic times using a comprehensive dataset of calyx biovolumes for 1005 crinoid genera (Supplementary Data 1), and explores the role of mass extinctions and global temperature in shaping these patterns.

Our analyses demonstrate that temporal variation in crinoid calyx size is scale-dependent; i.e., it is related to the considered taxonomic level and temporal duration of sequences. Remarkably, two major declines in body size at around the late Ordovician and the late Devonian extinction events are both evident, corroborating the view that mass extinction may considerably influence body size evolution^14^.

## Results and discussion

**Figure 1:**
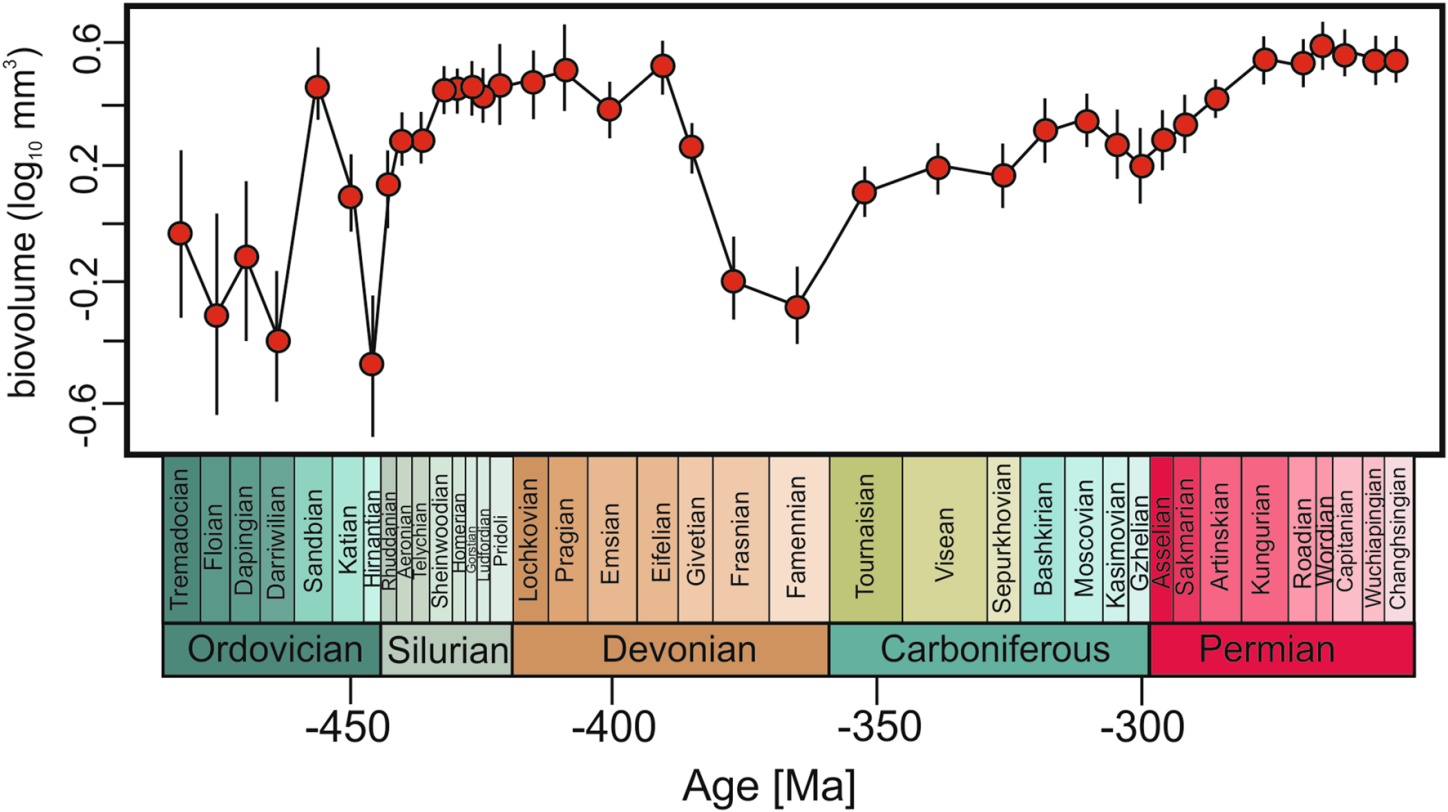
Trend in mean calyx size of crinoids as a whole in the Palaeozoic. Vertical lines represent error bars.

**Figure 2:**
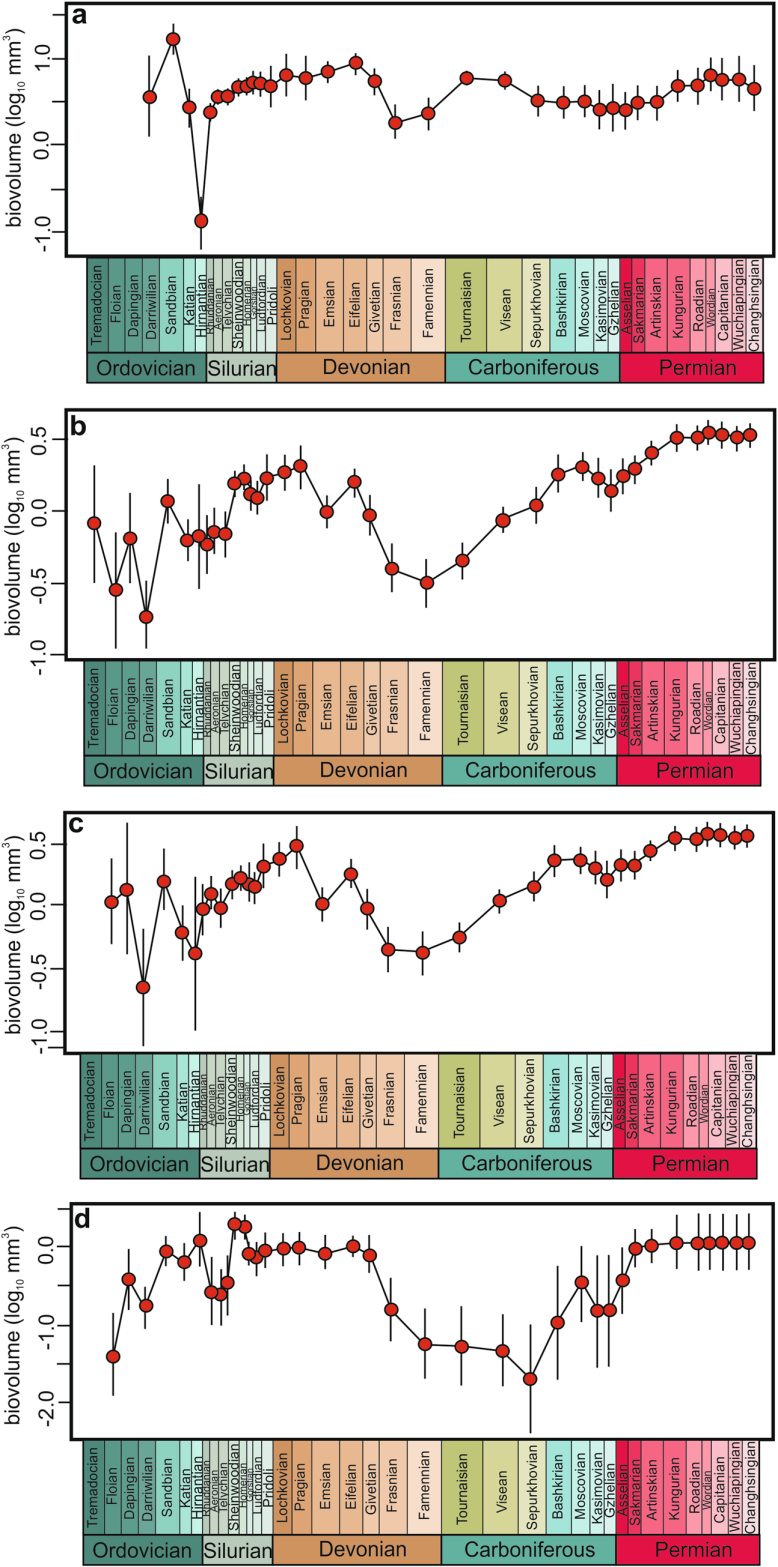
Trends in mean calyx size of major crinoid subclades in the Palaeozoic. **(a)** Camerata, **(b)** Pentacrinoidea, **(c)** Disparida, **(d)** Cladida. Vertical lines represent error bars.

**Figure 3:**
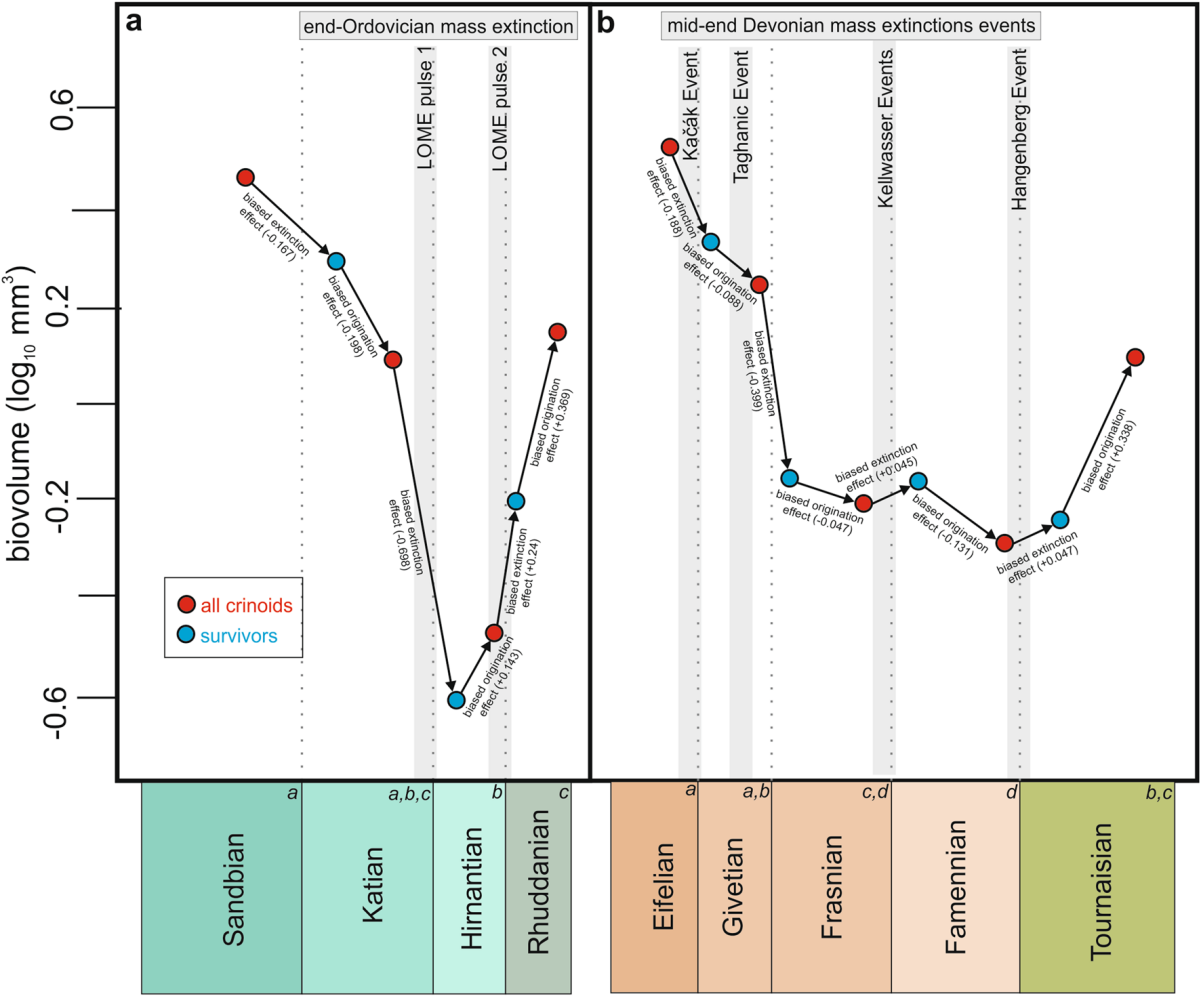
Plots explaining the calculation of the two major component values (biased extinction, biased origination). Interpretation of these plots works as follows, using the late Katian extinction as an example **(a)** and the late Givetian extinction as an example **(b)**. **(a)** In the Katian, 55 genera were extant; their mean biovolume size was 0.0874 log mm^3^. Of these 55 genera, 43 went extinct in this stage (with the mean size 0.282 log mm^3^), and 12 survived into the Hirnantian (the mean size of these −0.611 log mm^3^). Therefore, on average the survivors were smaller than the extinction victims. Thus, extinction was size-biased (larger genera were more likely to go extinct). The change in mean size due to this size-biased extinction was −0.611 log mm^3^—0.0874 log mm^3^ = − 0.6984 log mm^3^. In the Hirnantian, only 4 new genera originated with mean size −0.042 log mm^3^. The mean size in the Hirnantian of all 16 genera (12 survivors from the Katian and 4 originators) is −0.468 log mm^3^. Thus the 4 new originators changed the mean size of genera extant in the Hirnantian from −0.611 log mm^3^ to −0.468 log mm^3^ (a difference of 0.143 log mm^3^). In summary, the mean size of Katian taxa is 0.0874 log mm^3^, and the mean size of Hirnantian taxa is −0.468 log mm^3^, a change of −0.555 log mm^3^. This change of -0.555 log mm^3^ is partitioned into a size-biased extinction component of −0.6984 log mm^3^. Origination of new genera during the Hirnantian with mean size 0.143 log mm^3^ slightly mitigated the size decrease imposed by extinction component. Note that −0.6984 – (−0.143) = − 0.555. (**b**) In the Givetian, 75 genera were extant with the mean biovolume 0.254 log mm^3^. Of these 75 genera, 55 went extinct in this stage (with the mean size 0.4 log mm^3^), and 20 survived into the Frasnian (with the mean size −0.145 log mm^3^). Thus, extinction was size-biased (larger genera preferentially went extinct). The change in mean size due to this size-biased extinction was −0.145 log mm^3^ −0.254 log mm^3^ = 0.399 log mm^3^. In the Frasnian, 46 new genera originated with mean size -0.213 log mm^3^. The mean size in the Frasian of all 66 genera (20 survivors from the Givetian and 46 originators) is −0.192 log mm^3^. Thus the 46 new originators changed the mean size of genera extant in the Frasnian from −0.145 log mm^3^ to −0.192 log mm^3^ (a difference of 0.047 log mm^3^). In summary, the mean size of Givetian taxa is 0.254 log mm^3^, and the mean size of Frasian taxa is -0.192 log mm^3^, a change of −0.446 log mm^3^. This change of −0.446 log mm^3^ is partitioned into a size-biased extinction component of −0.399 log mm^3^ and a size-biased origination component of −0.047 log mm^3^. Note that −0.399−0.047 = −0.446. Periods sharing the same superscript indicate that size distributions are not significantly different from each other (p > 0.05).

At the class level, Crinoidea exhibit a heterogenous body size trajectory that is best fit by the unbiased random walk (URW) (Fig. 1, Table 1). The mean size of calyx had significantly fluctuated during the initial early Palaeozoic crinoid radiation. In the Ordovician it clearly shows an upward-downward trend with a peak in the early Late Ordovician (Sandbian, ~ 468 Ma). The first major decrease in mean (and maximum, see Supplementary Fig. S1) calyx size took place subsequently—in the Late Ordovician (Katian-Hirnantian), which roughly coincides with the Late Ordovician mass extinction, consisting of two extinction pulses initiated in the late Katian^15^. During this interval (especially during the Katian), crinoids had experienced a mass extinction and a major transition from the so-called Early Palaeozoic Crinoid Evolutionary Faunas (EPCEFs) mostly represented by disparids, diplobathrid camerates and hybocrinids to the Middle Palaeozoic Crinoid Evolutionary Faunas (MPCEFs) dominated by monobathrid camerates^16,17^. As shown herein, this extinction appears to be size-biased and the observed size decline is mainly governed by extinction of larger camerates (see Figs. 2, 3; Supplementary Table and Fig. S2). Intrestingly, this decline coincides with a major drop in the crinoid disparity and generic biodiversity^7^. Notably, this decline in calyx size was not driven by a long-term directional trend with a negative mean step change beginning in the pre-extinction intervals. Instead, the non-directional models (stasis or URW, depending on the scale) best fit the data (Table 1, Supplementary Tables and Figs. S1-S5), which is consistent with what can be predicted from geologically abrupt perturbation^18^. By contrast, disparids experienced a marked calyx size decline (also in the minimum size) after the Late Ordovician mass extinction (Fig. 2d, Supplementary Table and Fig. S4), which is more consistent with the so-called “Lilliput effect”^19^. Noteworthy, previous studies showed that some crinoids from Laurentia and Baltica at around the Late Ordovician extinction had experienced a significant reduction in body size that was ascribed to the “Lilliput Effect”^13^. Thus, these local trends can also be evidenced and observed at the global scale, despite uneven time-bin durations and low temporal resolution of our analyses. During the Silurian, the mean calyx size rebounded to pre-extinction dimensions and stabilised for a long (~ 50 Myrs) period until the Middle Devonian (Givetian, ~ 385 Ma), when the mean calyx size underwent an essentially continual decline until the end-Devonian (Fig. 1). Noticeably, this decline coincides with a drop in crinoid diversity and disparity^6,7^, and took place during a period encompassing a series of extinction pulses associated with anoxic events, spreading over ~ 25 Myrs^20,21^ (Fig. 3). Similarly, likewise during the end-Ordovician, this extinction is mostly size-biased (larger genera were more likely to go extinct, see Fig. 3). Likewise, the observed size decline is mainly governed by camerates (for which decrease in both minimum and maximum size is also observed; see Supplementary Table and Fig. S2) and to a lesser extent, by cladids (Fig. 2c, Supplementary Table and Fig. S5). Also, this decline was not part of an observable long-term pre-extinction negative driven trend (Table 1). Following the Devonian-Carboniferous boundary corresponding to the so-called Hangenberg event marking the last spike in the Devonian extinctions, the mean size increased stochastically throughout the rest of the Palaeozoic (Fig. 1), despite high taxonomic and environmental volatility (including during the Serpukhovian Biotic Crisis).

**Table 1:** Support (AICc weights) for models of calyx size evolution for crinoids as a whole and within major sister subclades for different intervals.

Model	Palaeozoic				
	All genera	Camerata	Pentacrinoidea	Disparida	Cladida
StrictStasis	0.000	0.007	0.000	0.002	0.000
Stasis	0.000	0.069	0.000	0.003	0.000
URW	**0.349**	0.018	0.187	0.284	0.148
GRW	0.168	0.005	0.121	0.102	0.080
Punc-1	0.000	0.016	0.000	0.002	0.000
Stasis-URW	0.023	0.076	0.057	0.065	0.036
Stasis-GRW	0.153	0.090	**0.414** (Tournaisian)	0.017	**0.575** (Tournaisian)
URW-Stasis	0.240	**0.582** (Telychian)	0.414	**0.422** (Asselian)	0.127
GRW-Stasis	0.066	0.135	0.048	0.103	0.033

	Carboniferous-Permian				
	All genera	Camerata	Pentacrinoidea	Disparida	Cladida
StrictStasis	0.000	**0.584**	0.000	0.002	0.000
Stasis	0.000	0.156	0.000	0.001	0.000
URW	0.021	0.201	0.013	0.066	0.017
GRW	**0.954**	0.045	**0.982**	**0.619**	**0.981**
Punc-1	0.021	0.006	0.000	0.273	0.000
Stasis-URW	0.001	0.001	0.000	0.001	0.000
Stasis-GRW	0.002	0.001	0.000	0.000	0.000
URW-Stasis	0.001	0.003	0.001	0.031	0.002
GRW-Stasis	0.001	0.002	0.004	0.008	0.000

	Silurian-mid Devonian				
	All genera	Camerata	Pentacrinoidea	Disparida	Cladida
GRW	0.252	**0.840**	0.051	0.024	0.054
URW	0.238	0.140	0.128	0.153	0.149
Stasis	0.096	0.007	0.157	0.160	0.149
StrictStasis	**0.414**	0.013	**0.663**	**0.663**	**0.647**

Best supported models are indicated in bold. For shorter intervals only simple models were taken into account. Time (geological period) of the shift in the evolutionary dynamics in the complex models are indicated in brackets.

Given that short-term and/or low taxonomic-level patterns may be masked over extended time periods or at high phylogenetic levels^22^, statistical fitting was also conducted at lower taxonomic levels and/or shorter time spans (encompassing intervals in between mass extinctions) (Table 1; Fig. 2; Supplementary Tables and Figs. S6-S15). At the lower taxonomic scale, contrasting patterns in body size dynamics of two major crinoid clades (Camerata and Pentacrinoidea) are evident, highlighting the intrinsic ecological and physiological differences between both groups (Fig. 2). Both clades revealed non-uniform complex body size dynamics with shifts (Table 1). Camerates display random size changes during the early Palaeozoic followed by a long period (~ 180 Myrs) of stasis for the remaining Palaeozoic times, notwithstanding high taxonomic and environmental volatility. In pentacrinoids, which are on average smaller that camerates, a transition in the evolutionary mode from a long period (~ 120 Myrs) of stasis to a driven positive trend following the late Devonian mass extinctions, is observed. Intriguingly, at shorter time intervals (in periods between mass extintions) opposite trends are visible in both clades (Supplementary Tables and Figs. S6-S15). During the Silurian—mid-Devonian time span, camerates reveal a positive directional trend, whereas body size trend of pentacrinoids (including cladids and disparids) is best described as strict stasis. These trends are reversed following the end-Devonian mass extinction, i.e., the mean body size of camerates appears stable, whereas a positive directional trend is observed in pentacrinoids (including cladids and disparids) (Table 1).

**Figure 4:**
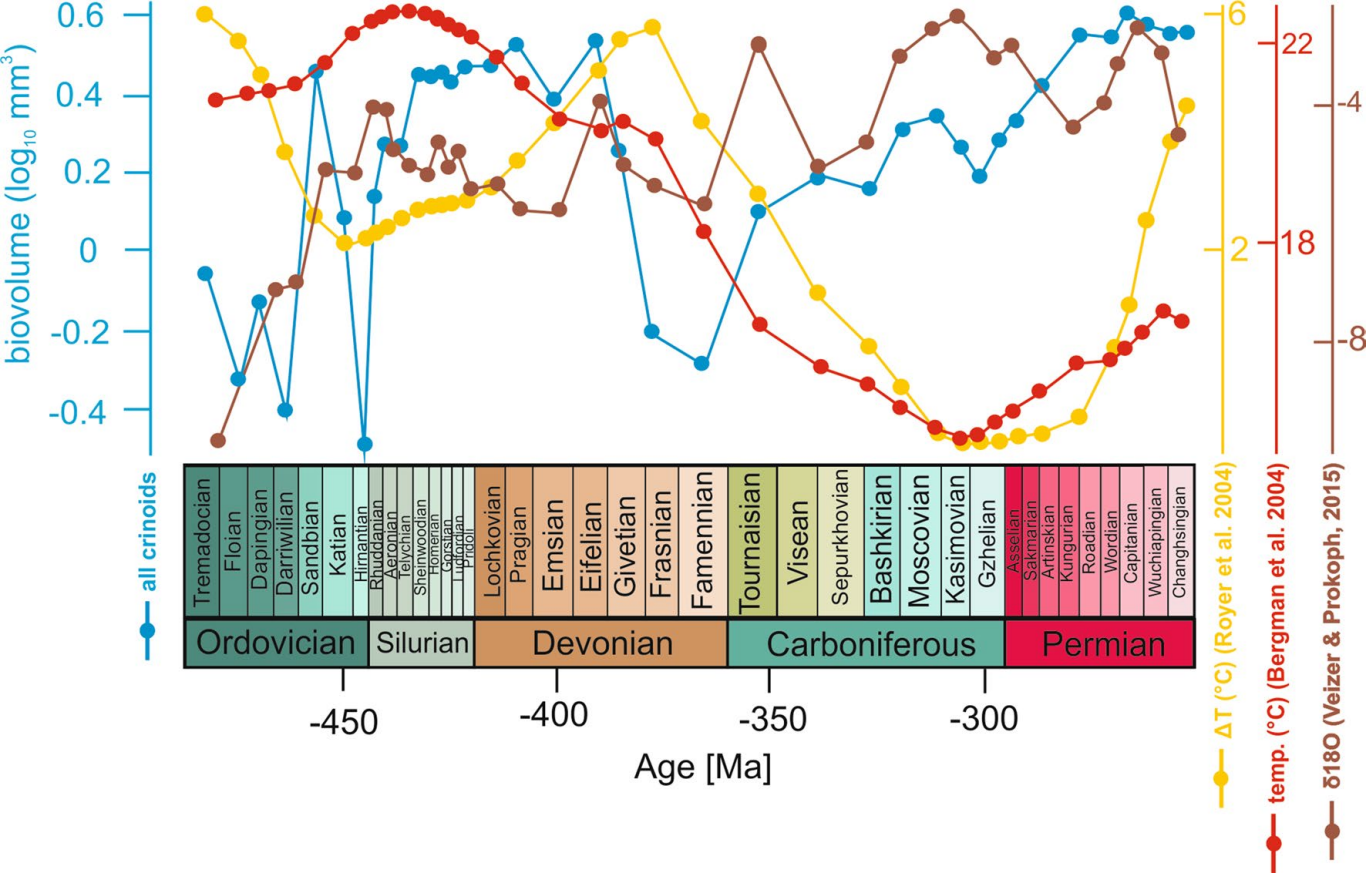
Mean calyx size of crinoids as a whole in the Palaeozoic plotted with temperature and δ18O curves inferred from three different sources.

Identification of the most important factors shaping these body size patterns is challenging. Temperature was commonly invoked as an important driver for body size evolution^23,24^. However, we found no relationship between crinoid body size and temperature (Supplementary Data 2). Although visual inspection and comparative analysis suggest that relationship between crinoid body size and global temperature may exist (Fig. 4), the correlation was not confirmed after detrending. Thus, given the marked complexity of the observed trends that differ at various taxonomic and temporal scales, it seems that a complex network of interrelated abiotic and biological factors might have affected body size evolution of Palaeozoic crinoids.

In addition to major climatic disruptions associated with extinction events, leading to shrinking body size, external ecological factors (such as predation pressure) might have contributed to some of the observed trends. For instance, in camerates a driven positive trend in the Silurian-Devonian and stasis in the post-Devonian times seems to mirror a pattern of increasing predation-resistant arm morphologies from the Ordovician through the Devonian, with no significant change in predation resistance following the end-Devonian Hangenberg extinction^25^ during which many predatory fish with grazing abilities disappeared^10^. Thus, predation pressure, which has been shown to be higher for camerates than for non-camerates^25^, might have been responsible for increasing theca size during the early-mid Palaeozoic. On the other hand, pentacrinoids, a major group in post-Devonian times, exhibit a driven trend in body size following the end-Devonian extinction events, which coincides with their morphological and phylogenetic diversification. The steady rise in body size of these crinoids, despite some taxonomic and environmental volatility (including unstable, fluctuating environmental conditions during the Late Palaeozoic Ice Age) may in part be also driven by the newly evolving Mississippian-style durophagous predators^2,10^.

The above data clearly show that crinoids exhibited a complex body size trajectory. Depending on taxonomic levels and temporal scales varying trends within Crinoidea are observed. These heterogenous dynamics might have been affected by a complex network of interacting physical and biological factors, rather than by a single driver (such as temperature). However, shared trends of shrinking body size at around the mass extinction events suggest that crinoid populations were responding similarly to broad-scale abrupt climatic perturbations. If the past can be used to illuminate the future, we may expect similar shrinking body size. Indeed, many species already exhibit smaller size as a result of climate change and many experimental studies suggest that a wide range of organisms, including echinoderms, may respond to unfavourable acidification and deoxygenation either by shrinking in size or by growing at a slower pace^26–28^.

## Methods

We compiled the respective calyx biovolumes of 1,005 crinoid genera spanning Ordovician–Permian interval into a database (details in Supplementary Data 1). The calyx is the most important body part containing most of the visceral organs and tissues. Importantly, this morphological part usually displays high fossilisation potential and is of taxonomic importance.

Biovolumes of respective calyces were estimated from the holotypes figured for type species mostly published in the Treatise on Invertebrate Paleontology. However, we also used primary literature when illustrations from the Treatise were insufficient to estimate the biovolumes. Recently described taxa (not included in Treatise) were also measured from figures in source papers. Calyx biovolume was approximated with standard volume calculations for different geometric solids following Brom et al.^12^ and Brom^29^. Our database contains 92% of all described Palaeozoic crinoid genera (cf., the newest crinoid database by Webster and Webster^30^ with updates to stratigraphic ranges^6^) supplemented with more recent taxonomic papers and revisions. Remaining named genera are either poorly illustrated or incompletely preserved.

Our database comprises log-transformed biovolumes assigned to their respective geological stages. We included one biovolume estimate for the entire stratigraphic range of a respective genus given that size of the holotype of type species is usually representative for this genus throughout its duration^3,29^. Our dataset was subjected to a time-series analysis using the “paleoTS” package with joint parametrization (v. 0.5.2; Hunt^31^) in Rstudio (R version 1.2.5033; R Core Team 2020^32^) in order to fit different likelihood models of trait evolution of the time-binned data^24^. The paleoTS package includes several simple (directional/general random walk, unbiased random walk, stasis, strict stasis) and complex, shift-including (punctuation, stasis-unbiased random walk, stasis-directional, unbiased random walk-stasis, directional-stasis) models. We compared support of these models using the ‟fit9models” function based on the small-sample corrected Akaike information criterion (AICc) and the Akaike weight. To test whether the support for each model can be related to temporal duration of sequences, and if different evolutionary models might characterize specific (and shorter) time intervals, we also performed model fitting on two ‛pruned’ datasets encompassing data from the intervals in between mass extinctions.

Analyses were also made between sister lineages (at the subclasses and parvclasses levels), which are nested at different taxonomic levels to determine which (if any) clade(s) are driving the overall trend and/or if any clades reveal dynamics that differ from the predominant pattern among the class Crinoidea.

In order to determine the role of climate factors in shaping body size trends, we sampled temperature and oxygen values from published datasets. As multiple estimates from various models and proxies exist, we sampled values independently from three different sources (“COPSE” model temperature estimates (Fig. 5 in^33^), red curve from Fig 4 in^34^, and δ^18^O data (inverse proxy for temperature) (Fig. 5 in^35^)). The effect of temperature on body size was analysed using the GLS (generalised least-squares) regression models in R using the “nlme” package (v. 3.1-143^36^), both with a first-order autoregressive model (AR1) to eliminate autocorrelation, and with no autoregressive model (AR0)^37^. All models were compared through AICc in pairs: one considering temperature as a predictor, and one assuming no such relation (null model). These analyses were performed at various taxonomic ranks (for the entire class and for constituent clades (subclasses: Camerata vs. Pentacrinoidea and parvclasses: Cladida vs. Disparida) and were also conducted separately for time series spanning shorter time intervals.

The probability that the body size from the pre-extinction, extinction and post-extinction intervals were drawn from equivalent distributions, was tested using a Kolmogorov–Smirnov (K-S) tests. The effects of extinction and origination on the overall size distribution was assessed following Rego et al.^38^ and Zhang et al.^39^.

## Supplementary Information

Supplementary Information 1.

Supplementary Information 2.

Supplementary Information 3.

## Data Availability

Most data are available in the main text and in the supplementary materials. R script and source files are available in: https://osf.io/m3gt5/.
