# Supplementary material for: Shared patterns in body size declines among crinoids during the Palaeozoic extinction events
Source: Sci Rep. 2021 Oct 13;11:20351. doi: 10.1038/s41598-021-99789-6 (PMC8514529; doi:10.1038/s41598-021-99789-6)

Supplementary Tables and Figures for

Shared patterns in body size declines among crinoids during the Palaeozoic extinction events

Mariusz A. Salamon, Tomasz Brachaniec, Dorota Kołbuk, Anwesha Saha, Przemysław Gorzelak*

*Corresponding author. Email:pgorzelak@twarda.pan.pl

This PDF file includes:

Figs. S1 to S15
Tables S1 to S15

Other Supplementary Materials for this manuscript include the following:

Data S1. Calyx biovolumes of holotypes of type species of Paleozoic crinoids.
Data S2. Results of correletions between body size and temperature.

Supplementary Table. 1. Comparison of support for each model of calyx size evolution for crinoids as a whole. AICC values and Akaike weights, LogL= model log likelihood, K = number of model parameters, delta AICc.

	logL	K	AICc	dAICc	Akaike.wt
StrictStasis	-29.6022399	1	61.3159087	78.7040382	0.000
Stasis	-1.643132	2	7.6291212	25.0172507	0.000
URW	10.865493	2	-17.3881295	0.0000000	**0.349**
GRW	11.316337	3	-15.9267910	1.4613384	0.168
Punc-1	5.043251	4	-0.8743811	16.5137483	0.000
Stasis-URW	10.600193	4	-11.9882655	5.3998639	0.023
Stasis-GRW	13.804554	5	-15.7341077	1.6540218	0.153
URW-Stasis	14.254105	5	-16.6332108	0.7549187	0.240
GRW-Stasis	14.385930	6	-14.0621823	3.3259472	0.066

Supplementary Fig. 1. Distribution of maximum, mean and minimum biovolumes for all Paleozoic crinoids. Note that maximum values reveal a trend of increasing and decreasing towards a peak in the mid-Devonian. The distribution of minimum shows considerable instability through much of the early Paleozoic. Then, it shows some longer periods of stability: highs in the Silurian and early Devonian, and lows in the mid-Devonian–early Permian.

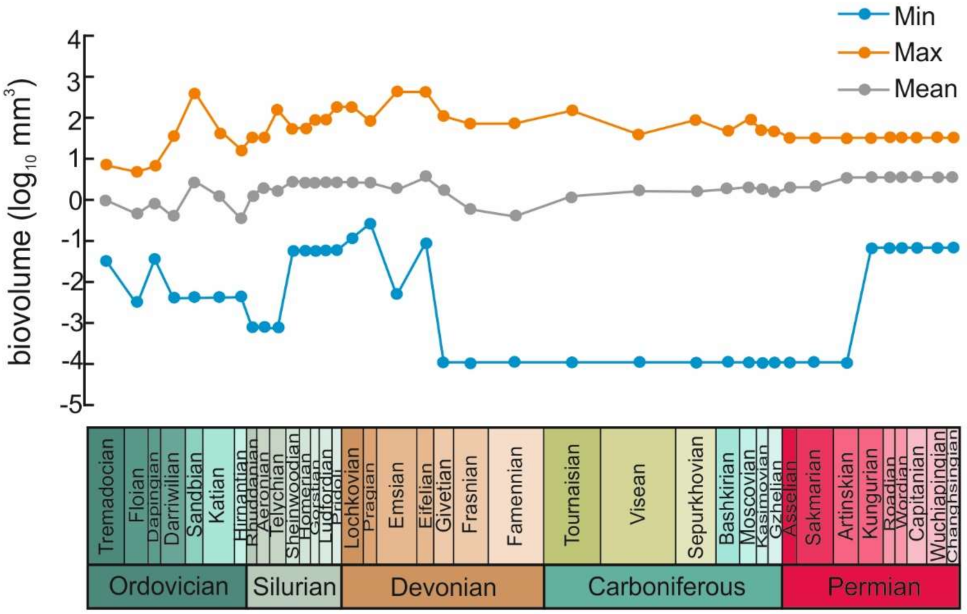

Supplementary Table. 2. Comparison of support for each model of calyx size evolution for Camerata. AICC values and Akaike weights, LogL= model log likelihood, K = number of model parameters, delta AICc. Shift: Telychian.

	logL	K	AICc	dAICc	Akaike.wt
StrictStasis	-6.2946999	1	14.710612	8.807218	0.007
Stasis	-2.8929724	2	10.160945	4.257551	0.069
URW	-4.2377664	2	12.850533	6.947139	0.018
GRW	-4.2310155	3	15.236224	9.332831	0.005
Punc-1	-1.8601129	4	13.053559	7.150166	0.016
Stasis-URW	-0.3164076	4	9.966149	4.062755	0.076
Stasis-GRW	1.2219514	5	9.625063	3.721669	0.090
URW-Stasis	3.0827861	5	5.903393	0.000000	**0.582**
GRW-Stasis	3.0892551	6	8.821490	2.918096	0.135

Supplementary Fig. 2. Distribution of maximum, mean and minimum biovolumes for camerate crinoids. Note that maximum values reveal some fluctuations but show a general trend of increasing and decreasing towards a peak in the mid-Devonian. The distribution of minimum shows some periods with stability (Silurian and mid-Carboniferous–early Permian). Note that two extinctions events are generally accompanied by declines in maximum, mean and minimum biovolumes.

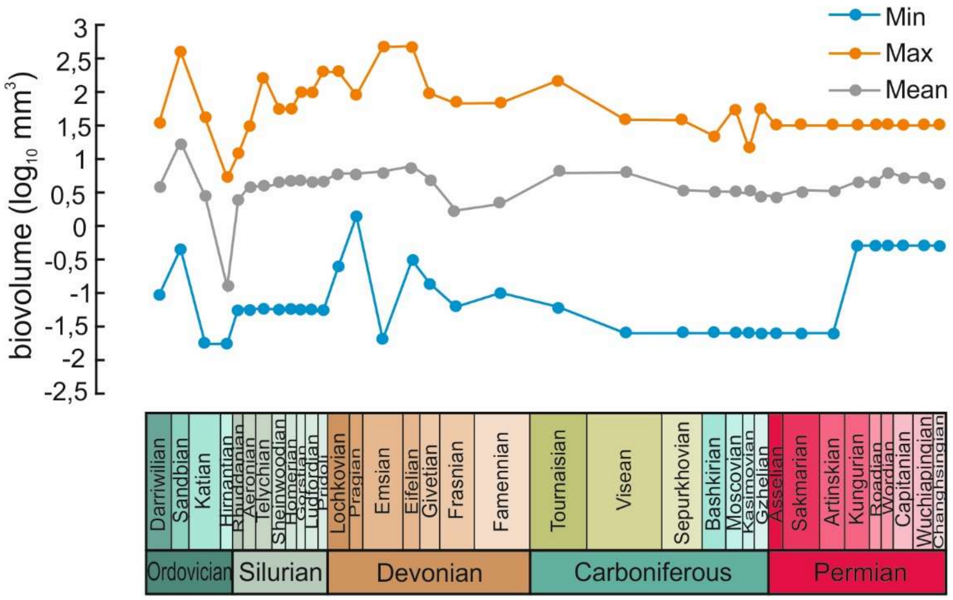

Supplementary Table. 3. Comparison of support for each model of calyx size evolution for Pentacrinoidea. AICC values and Akaike weights, LogL= model log likelihood, K = number of model parameters, delta AICc. Shift: Tournaisian.

	logL	K	AICc	dAICc	Akaike.wt
StrictStasis	-69.321635	1	140.754381	157.545084	0.000
Stasis	-9.645200	2	23.633257	40.423960	0.000
URW	9.773089	2	-15.203320	1.587383	0.187
GRW	10.514830	3	-14.323777	2.466926	0.121
Punc-1	3.683316	4	1.845488	18.636191	0.000
Stasis-URW	11.019369	4	-12.826617	3.964085	0.057
Stasis-GRW	14.332851	5	-16.790703	0.000000	**0.414**
URW-Stasis	13.462732	5	-15.050464	1.740239	0.173
GRW-Stasis	13.587828	6	-12.465979	4.324724	0.048

Supplementary Fig. 3. Distribution of maximum, mean and minimum biovolumes for Pentacrinoidea. Note that maximum values are overall very stable but the distribution of minimum shows considerable instability through much of the early-mid Paleozoic followed by two longer periods with stability.

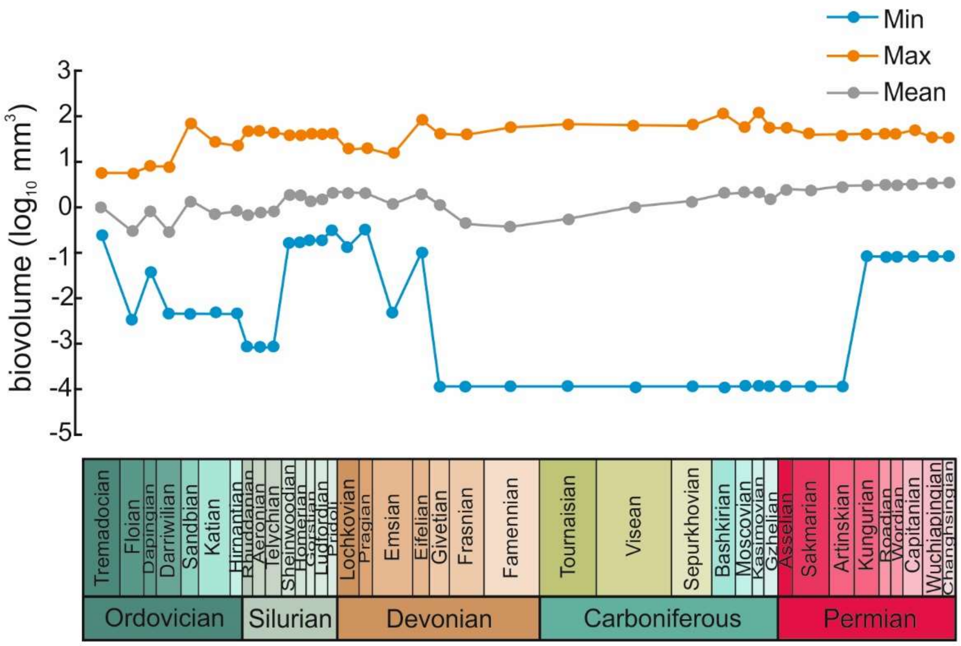

Supplementary Table. 4. Comparison of support for each model of calyx size evolution for Disparida. AICC values and Akaike weights, LogL= model log likelihood, K = number of model parameters, delta AICc. Shift: Asselian.

	logL	K	AICc	dAICc	Akaike.wt
StrictStasis	-23.22346	1	48.56120	10.9397109	0.002
Stasis	-21.66797	2	47.68888	10.0673956	0.003
URW	-17.03206	2	38.41705	0.7955675	0.284
GRW	-16.86731	3	40.46190	2.8404088	0.102
Punc-1	-19.31664	4	47.88329	10.2618010	0.002
Stasis-URW	-16.05265	4	41.35529	3.7338049	0.065
Stasis-GRW	-16.04337	5	44.02222	6.4007383	0.017
URW-Stasis	-12.84300	5	37.62149	0.0000000	**0.422**
GRW-Stasis	-12.82056	6	40.44113	2.8196416	0.103

Supplementary Fig. 4. Distribution of maximum, mean and minimum biovolumes for disparid crinoids. Note that maximum and minimum values reveal heterogenous trajectories. Some notable declines in minimum size are observed after the end-Ordovician extinction and during and in the aftermath of the end-Devonian extinction. However, interpretation of evolutionary trend in disparids should be treated with caution as they persisted as a relatively minor clade into the Mississippian.

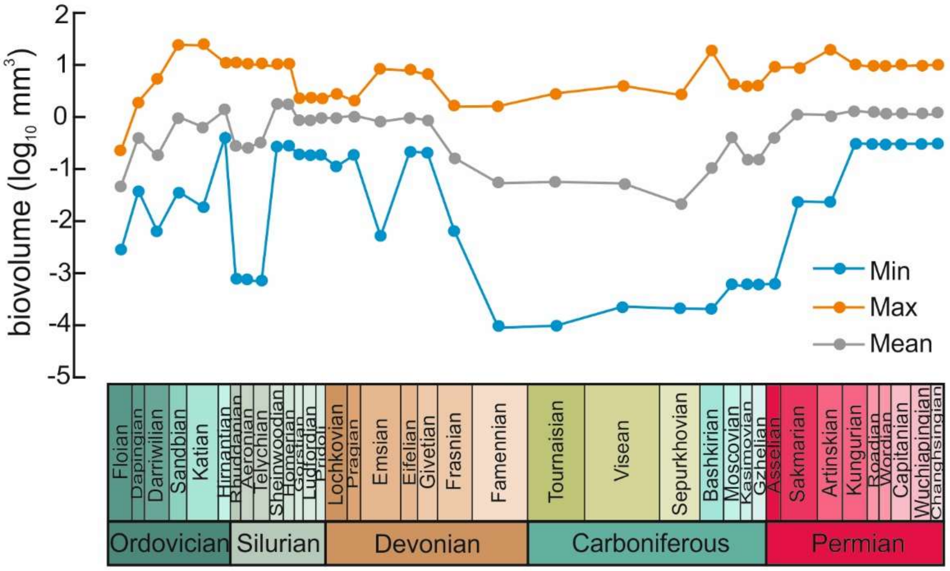

Supplementary Table. 5. Comparison of support for each model of calyx size evolution for Cladida. AICC values and Akaike weights, LogL= model log likelihood, K = number of model parameters, delta AICc. Shift: Tournaisian.

	logL	K	AICc	dAICc	Akaike.wt
GRW	-46.006101	1	94.126488	115.993426	0.000
URW	-5.468756	2	15.290453	37.157390	0.000
Stasis	11.756137	2	-19.159333	2.707605	0.148
StrictStasis	12.330786	3	-17.934300	3.932637	0.080
Punc-1	8.197242	4	-7.144484	14.722453	0.000
Stasis-URW	12.782062	4	-16.314125	5.552813	0.036
Stasis-GRW	16.901211	5	-21.866937	0.000000	**0.575**
URW-Stasis	15.390023	5	-18.844563	3.022375	0.127
GRW-Stasis	15.486589	6	-16.173179	5.693759	0.033

Supplementary Fig. 5. Distribution of maximum, mean and minimum biovolumes for cladid crinoids. Note that maximum values are overall very stable but the distribution of minimum shows considerable instability through much of the early-mid Paleozoic followed by two longer periods with stability.

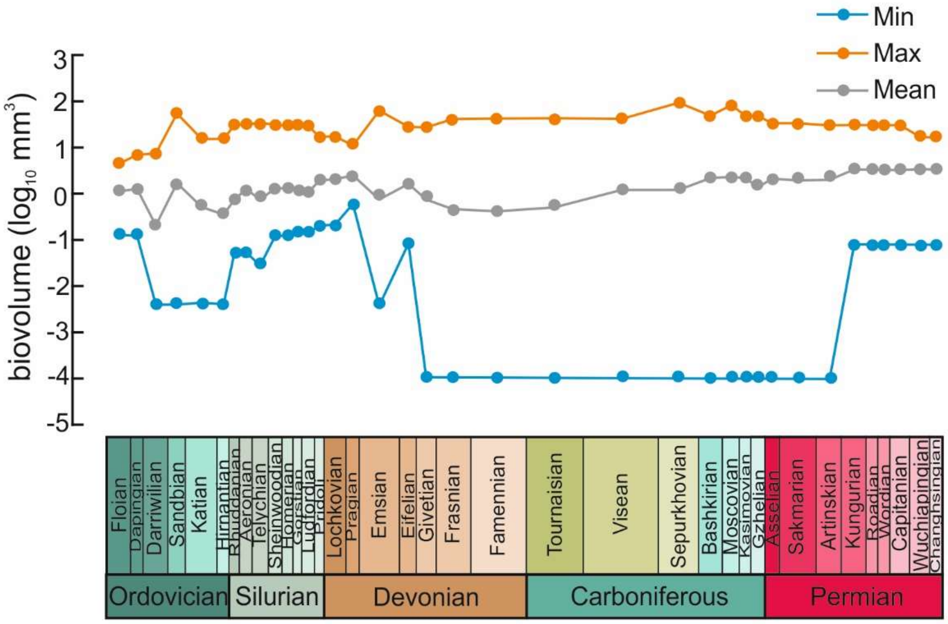

Supplementary Table. 6. Comparison of support for each model of calyx size evolution for crinoids as a whole in Silurian–mid-Devonian. AICC values and Akaike weights, LogL= model log likelihood, K = number of model parameters, delta AICc.

	logL	K	AICc	dAICc	Akaike.wt
GRW	12.780519	3	-16.56104	0.9894667	0.252
URW	10.886389	2	-16.43944	1.1110594	0.238
Stasis	9.982373	2	-14.63141	2.9190904	0.096
StrictStasis	9.975252	1	-17.55050	0.0000000	**0.414**

Supplementary Fig. 6. Trend in mean calyx size of crinoids as a whole in Silurian–mid-Devonian.

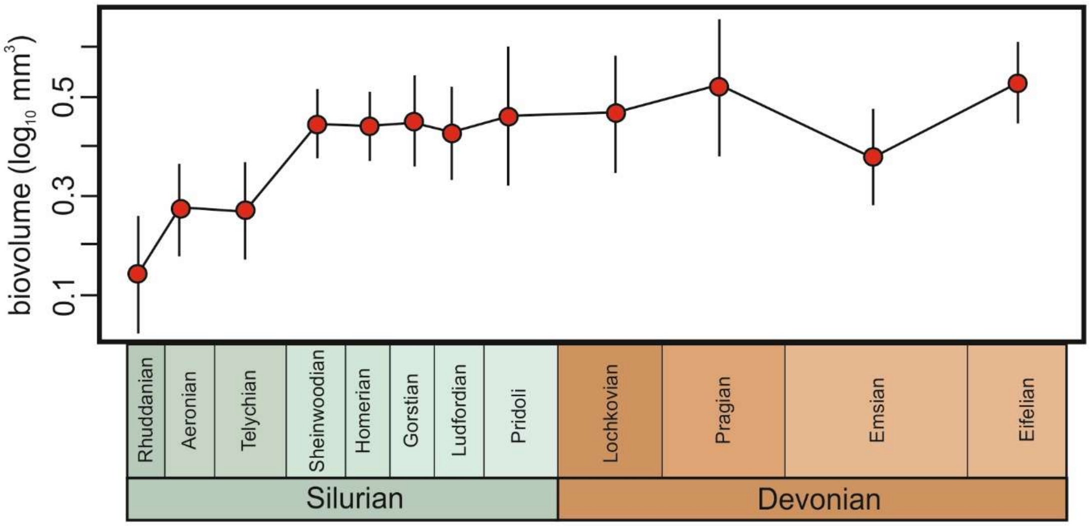

Supplementary Table. 7. Comparison of support for each model of calyx size evolution for Camerata in Silurian–mid-Devonian. AICC values and Akaike weights, LogL= model log likelihood, K = number of model parameters, delta AICc.

	logL	K	AICc	dAICc	Akaike.wt
GRW	11.787729	3	-14.575458	0.000000	**0.840**
URW	8.159024	2	-10.984715	3.590743	0.140
Stasis	5.129168	2	-4.925003	9.650455	0.007
StrictStasis	4.336511	1	-6.273023	8.302435	0.013

Supplementary Fig. 7. Trend in mean calyx size of Camerata in Silurian–mid-Devonian.

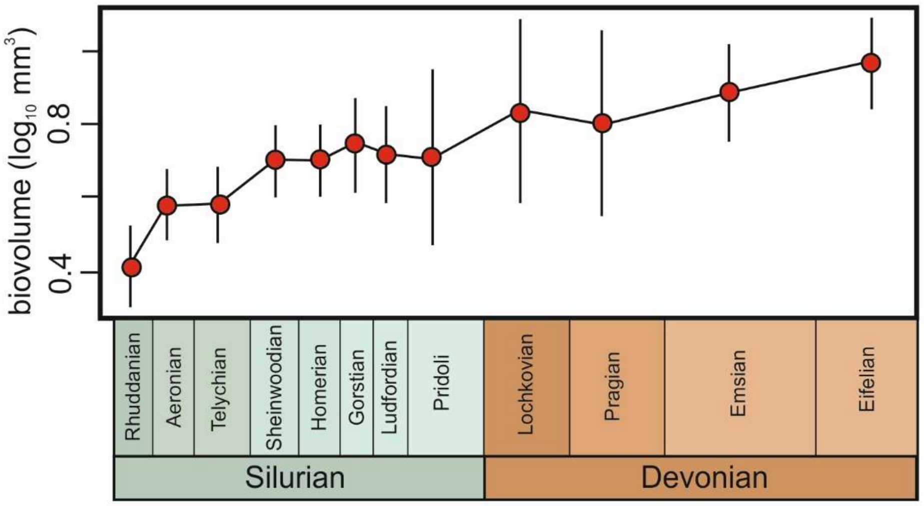

Supplementary Table. 8. Comparison of support for each model of calyx size evolution for Pentacrinoidea in Silurian–mid-Devonian. AICC values and Akaike weights, LogL= model log likelihood, K = number of model parameters, delta AICc.

	logL	K	AICc	dAICc	Akaike.wt
GRW	6.270544	3	-3.541088	5.115429	0.051
URW	5.350867	2	-5.368401	3.288116	0.128
Stasis	5.555972	2	-5.778612	2.877905	0.157
StrictStasis	5.528258	1	-8.656517	0.000000	**0.663**

Supplementary Fig. 8. Trend in mean calyx size of Pentacrinoidea in Silurian–mid-Devonian.

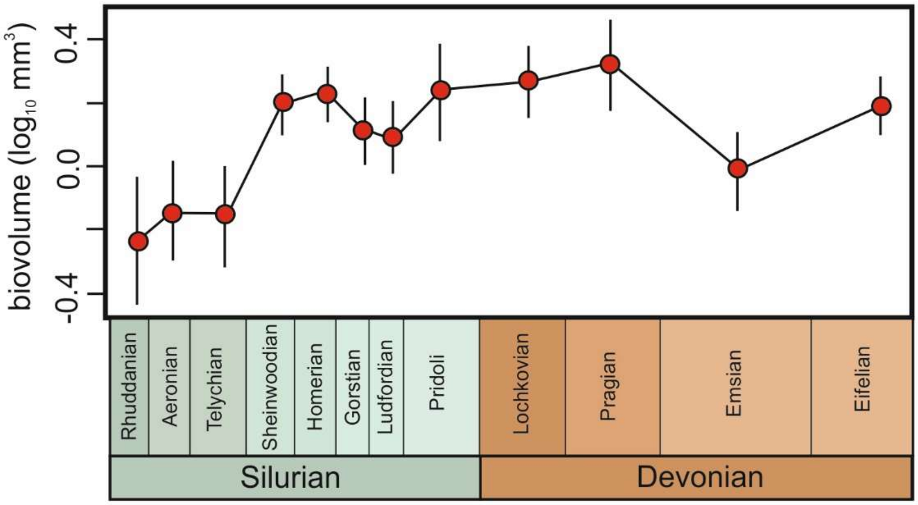

Supplementary Table. 9. Comparison of support for each model of calyx size evolution for Disparida in Silurian–mid-Devonian. AICC values and Akaike weights, LogL= model log likelihood, K = number of model parameters, delta AICc.

	logL	K	AICc	dAICc	Akaike.wt
GRW	0.5169082	3	7.966184	6.599037	0.024
URW	0.5164268	2	4.300480	2.933333	0.153
Stasis	0.5599399	2	4.213454	2.846307	0.160
StrictStasis	0.5164268	1	1.367146	0.000000	**0.663**

Supplementary Fig. 9. Trend in mean calyx size of Disparida in Silurian–mid-Devonian.

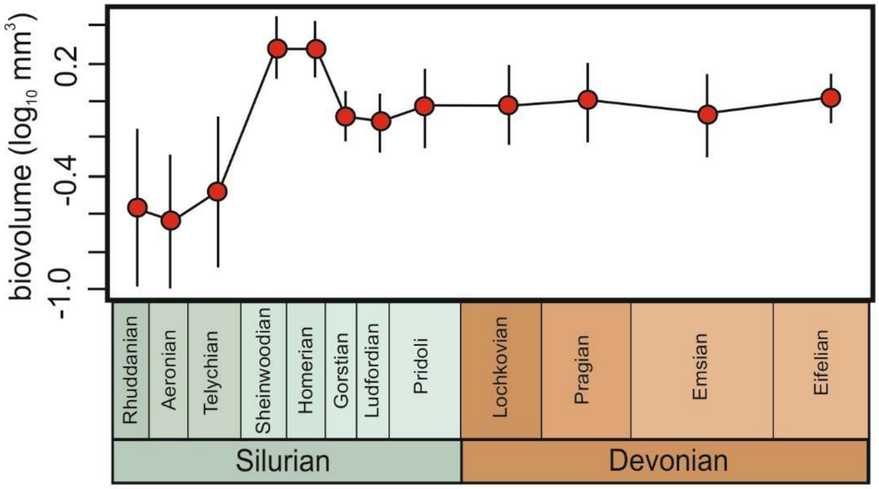

Supplementary Table. 10. Comparison of support for each model of calyx size evolution for Cladida in Silurian–mid-Devonian. AICC values and Akaike weights, LogL= model log likelihood, K = number of model parameters, delta AICc.

	logL	K	AICc	dAICc	Akaike.wt
GRW	8.004920	3	-7.009840	4.958878	0.054
URW	7.184359	2	-9.035385	2.933333	0.149
Stasis	7.184359	2	-9.035385	2.933333	0.149
StrictStasis	7.184359	1	-11.968718	0.000000	**0.647**

Supplementary Fig. 10. Trend in mean calyx size of Cladida in Silurian–mid-Devonian.

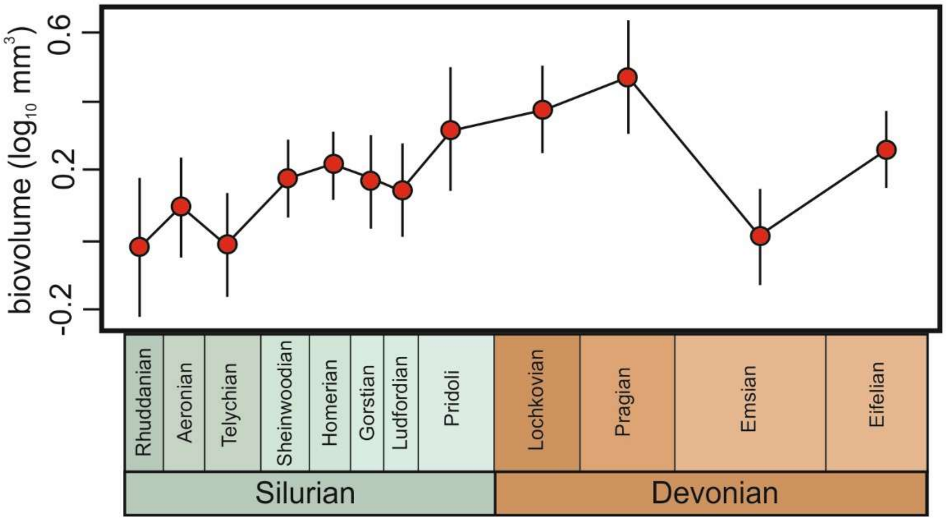

Supplementary Table. 11. Comparison of support for each model of calyx size evolution for crinoids as a whole in Carboniferous-Permian. AICC values and Akaike weights, LogL= model log likelihood, K = number of model parameters, delta AICc.

	logL	K	AICc	dAICc	Akaike.wt
StrictStasis	-4.542552	1	11.370818	45.155287	0.000
Stasis	6.342238	2	-7.761399	26.023071	0.000
URW	15.520152	2	-26.117227	7.667243	0.021
GRW	20.892235	3	-33.784470	0.000000	**0.954**
Punc-1	18.878854	4	-26.121344	7.663125	0.954
Stasis-URW	15.654740	4	-19.673117	14.111353	0.001
Stasis-GRW	18.562464	5	-21.124927	12.659543	0.002
URW-Stasis	17.431657	5	-18.863315	14.921155	0.001
GRW-Stasis	20.955339	6	-20.577345	13.207125	0.001

Supplementary Fig. 11. Trend in mean calyx size of crinoids as a whole in Carboniferous-Permian.

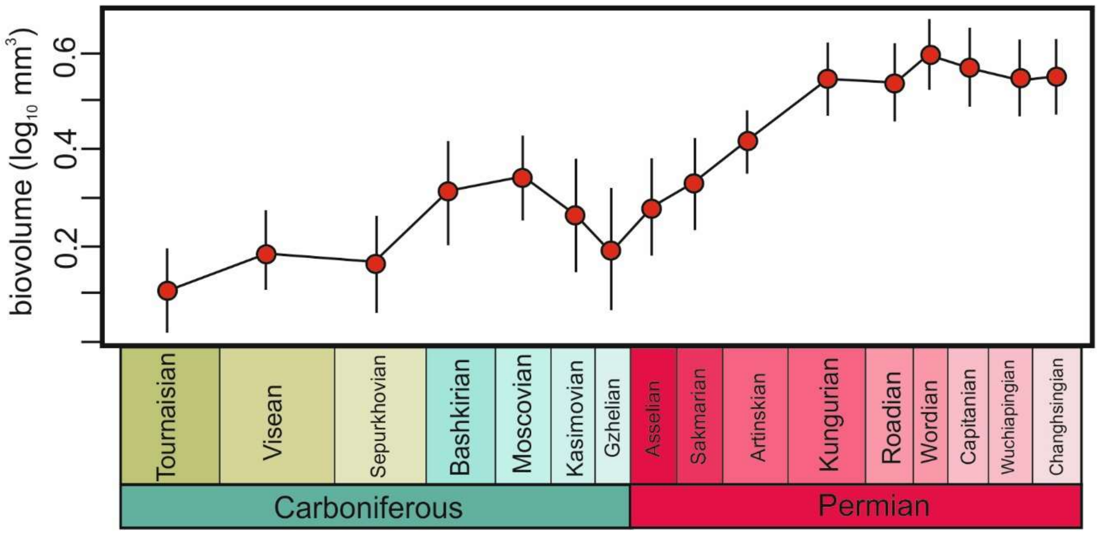

Supplementary Table. 12. Comparison of support for each model of calyx size evolution for Camerata in Carboniferous-Permian. AICC values and Akaike weights, LogL= model log likelihood, K = number of model parameters, delta AICc.

	logL	K	AICc	dAICc	Akaike.wt
StrictStasis	6.741642	1	-11.1975688	0.000000	**0.584**
Stasis	6.741642	2	-8.5602061	2.637363	0.156
URW	6.995804	2	-9.0685306	2.129038	0.201
GRW	7.042612	3	-6.0852244	5.112344	0.045
Punc-1	6.905081	4	-2.1737987	9.023770	0.006
Stasis-URW	5.282048	4	1.0722673	12.269836	0.001
Stasis-GRW	7.404315	5	1.1913691	12.388938	0.001
URW-Stasis	8.159457	5	-0.3189136	10.878655	0.003
GRW-Stasis	10.778289	6	-0.2232445	10.974324	0.002

Supplementary Fig. 12. Trend in mean calyx size of Camerata in Carboniferous-Permian.

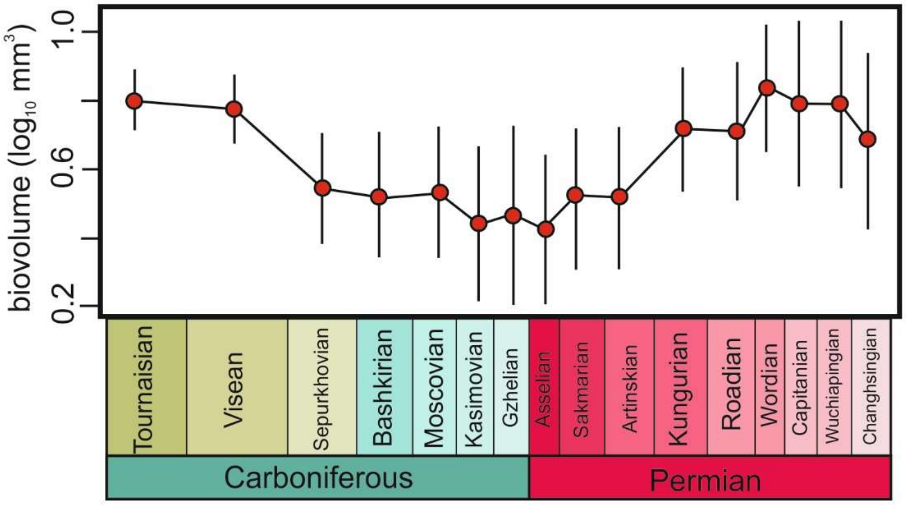

Supplementary Table. 13. Comparison of support for each model of calyx size evolution for Pentacrinoidea in Carboniferous-Permian. AICC values and Akaike weights, LogL= model log likelihood, K = number of model parameters, delta AICc.

	logL	K	AICc	dAICc	Akaike.wt
StrictStasis	-25.9317915	1	54.149297	80.032469	0.000
Stasis	-0.1181194	2	5.159316	31.042488	0.000
URW	11.0752551	2	-17.227433	8.655739	0.013
GRW	16.9415861	3	-25.883172	0.000000	**0.982**
Punc-1	7.7511220	4	-3.865880	22.017292	0.000
Stasis-URW	8.1770159	4	-4.717668	21.165504	0.000
Stasis-GRW	8.8238292	5	-1.647658	24.235514	0.000
URW-Stasis	14.3272328	5	-12.654466	13.228707	0.001
GRW-Stasis	18.1317070	6	-14.930081	10.953092	0.004

Supplementary Fig. 13. Trend in mean calyx size of Pentacrinoidea in Carboniferous-Permian.

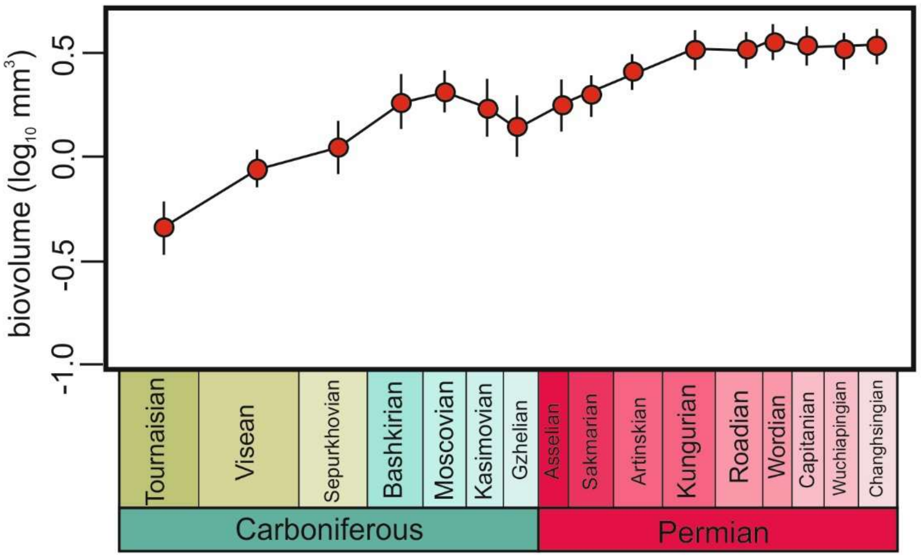

Supplementary Table. 14. Comparison of support for each model of calyx size evolution for Disparida in Carboniferous-Permian. AICC values and Akaike weights, LogL= model log likelihood, K = number of model parameters, delta AICc.

	logL	K	AICc	dAICc	Akaike.wt
StrictStasis	-12.804646	1	27.89501	11.448674	0.002
Stasis	-12.781726	2	30.48653	14.040198	0.001
URW	-8.005893	2	20.93486	4.488532	0.066
GRW	-4.223166	3	16.44633	0.000000	**0.619**
Punc-1	-3.224432	4	18.08523	1.638896	0.273
Stasis-URW	-8.848870	4	29.33410	12.887773	0.001
Stasis-GRW	-8.011602	5	32.02320	15.576874	0.000
URW-Stasis	-3.224434	5	22.44887	6.002536	0.031
GRW-Stasis	-1.935858	6	25.20505	8.758719	0.008

Supplementary Fig. 14. Trend in mean calyx size of Disparida in Carboniferous-Permian.

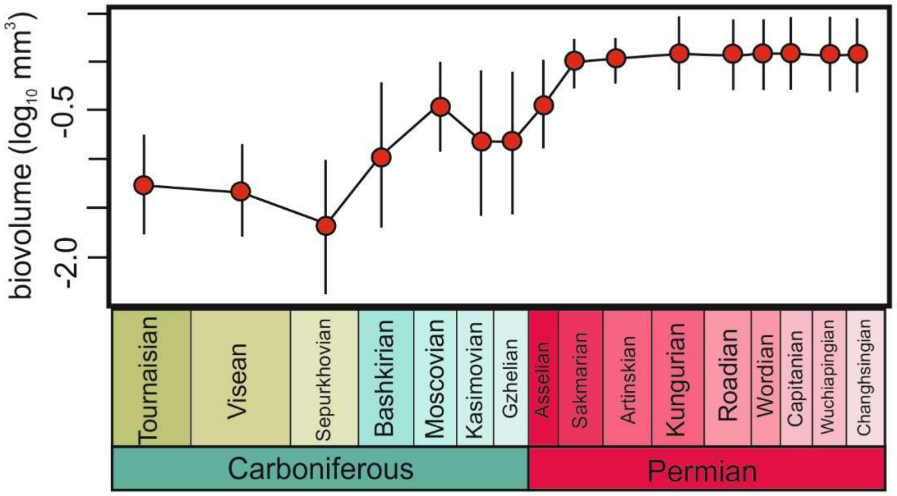

Supplementary Table. 15. Comparison of support for each model of calyx size evolution for Cladida in Carboniferous-Permian. AICC values and Akaike weights, LogL= model log likelihood, K = number of model parameters, delta AICc.

	logL	K	AICc	dAICc	Akaike.wt
StrictStasis	-19.372783	1	41.031280	67.49255	0.000
Stasis	1.547113	2	1.828852	28.29013	0.000
URW	11.626100	2	-18.329123	8.13215	0.017
GRW	17.230637	3	-26.461274	0.000000	**0.981**
Punc-1	8.807789	4	-5.979215	20.48206	0.000
Stasis-URW	8.920129	4	-6.203894	20.25738	0.000
Stasis-GRW	12.265501	5	-8.531002	17.93027	0.000
URW-Stasis	14.759916	5	-13.519831	12.94144	0.002
GRW-Stasis	16.313208	6	-11.293082	15.16819	0.000

Supplementary Fig. 15. Trend in mean calyx size of Cladida in Carboniferous-Permian.

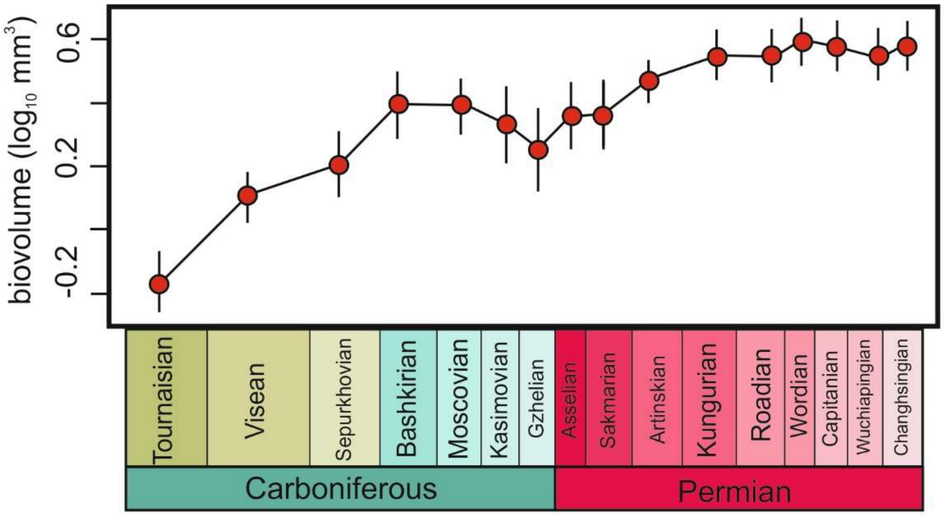

Supplement: Supplementary file 3 — Supplementary Information 3. [file 41598_2021_99789_MOESM3_ESM.pdf]
